# Beyond the *p* Value Dichotomy: Alternatives for Statistical Inference—A Critical Review

**DOI:** 10.1111/jep.70373

**Published:** 2026-02-04

**Authors:** Matheus Hissa Lourenço Ferreira, Lucas Caseri Câmara, Nelson Carvas Junior

**Affiliations:** ^1^ Federal University of Minas Gerais (UFMG) Belo Horizonte MG Brazil; ^2^ Department of Specialization in Clinical Anabolism, Brazilian Society of Endocrinology and Metabolism in Sports and Exercise, College of Governance Engineering, and Education of São Paulo (FGE‐SP) São Paulo Brazil; ^3^ Paulista University (UNIP) São Paulo SP Brazil; ^4^ Department of Postgraduate Studies in Evidence‐Based Health Federal University of São Paulo (UNIFESP) São Paulo SP Brazil

**Keywords:** Bayes theorem, clinical decision‐making, confidence intervals, data interpretation, evidence‐based practice, reproducibility of results, sports medicine, statistical

## Abstract

**Rationale:**

The *p* value has long been used as the primary criterion for statistical significance; however, its dichotomous interpretation has been increasingly criticized for oversimplifying uncertainty and distorting scientific inference, particularly in health and sports sciences.

**Aims and Objectives:**

This study aimed to critically analyze the limitations of using the *p* value as the central criterion of statistical significance and to discuss more robust methodological alternatives for statistical inference.

**Methods:**

A critical review was conducted using the PubMed/MEDLINE database covering the period from 2015 to 2025, complemented by citation tracking. Reviews, editorials, guidelines, and methodological essays that directly addressed the interpretation of *p* values and complementary metrics were included. A total of 46 articles were selected and evaluated using a self‐developed critical appraisal checklist.

**Results:**

Among the included studies, 38 (82.6%) explicitly criticized the isolated or dichotomous use of the *p* value, whereas eight adopted a more moderate position, supporting its use only when combined with confidence intervals, effect sizes, or Bayesian approaches. No article defended the *p* value as a standalone criterion for scientific decision‐making. The most frequent recommendations involved abandoning the term “statistically significant,” prioritizing the estimation of effect magnitude and precision, and promoting the use of compatibility intervals, effect sizes, and Bayesian methods.

**Conclusion:**

Overcoming the binary logic of *p* < 0.05 is essential to enhance transparency, reduce bias, and better align statistical practice with the scientific and clinical relevance of research findings, particularly in the health and sports sciences.

## 1 Introduction

Researchers in medicine and sports sciences rely on different methodological strategies to evaluate the strength of evidence regarding specific associations or effects. For decades, hypothesis testing has been the dominant framework, with the *p* value widely used to quantify evidence against a null hypothesis [1]. This practice, however, has consolidated into a dichotomous logic that classifies results as ‘significant’ or ‘non‐significant,’ usually anchored in the conventional threshold of *p* < 0.05.

Although deeply entrenched, this paradigm has important conceptual and practical limitations. The *p* value does not convey information about the magnitude or clinical relevance of an effect, and it is strongly influenced by sample size and data variability [2, 3]. As a result, trivial differences can reach statistical significance in large samples, while clinically meaningful effects may be dismissed in small studies. This binary interpretation reinforces an illusion of certainty and oversimplifies complex phenomena, contributing to the reproducibility crisis, especially in sports science, where small samples and fragile designs are common [2].

A particularly concerning issue is that statistical significance is often interpreted as evidence of stability and reliability. However, *p* values are inherently unstable and can fluctuate widely even under exact replications due solely to sampling variability [4]. For example, in identical replication attempts, a result can easily swing from highly significant (e.g., *p* < 0.001) to clearly non‐significant (e.g., *p* > 0.20) purely by chance, without any change in the underlying effect or study design. Empirical investigations in clinical and biomedical research further demonstrate that small data perturbations or minimal event changes can shift a result from ‘significant’ to ‘non‐significant,’ underscoring the fragility of conclusions based on hypothesis testing alone [5]. This intrinsic volatility reveals that statistical significance offers little assurance of robustness and can lead to misleading inferences, including the overestimation of trivial effects or the disregard of clinically meaningful findings. Such fragility undermines confidence in published results and highlights the need for inferential approaches that prioritize estimation, uncertainty, and replication.

The statistical community has increasingly called for reform. The 2016 statement from the American Statistical Association warned against misuse of the *p* value and highlighted that it should not be used as a sole arbiter of scientific validity [6]. More recently, an influential editorial in the New England Journal of Medicine recommended retiring the labels ‘statistically significant’ and ‘non‐significant,’ emphasizing that such terms are misleading shortcuts that distort interpretation. Instead, *p* values should be presented as continuous measures, interpreted alongside effect size estimates, compatibility (confidence) intervals, and other metrics of uncertainty. An additional refinement is hypothesis evaluation using an interval null, which assesses compatibility with a range of practically null effects rather than testing a point null, thereby avoiding dichotomous decisions based solely on *p* values.

A key step in moving beyond dichotomous inference is reframing research questions themselves. Rather than asking ‘do A and B differ?’, estimation thinking encourages researchers to ask ‘to what extent do A and B differ?’, thereby promoting quantitative answers grounded in effect sizes and measures of precision [4].

Effect sizes are particularly valuable, as they indicate the practical magnitude of an association or intervention. Two results may both reach *p* < 0.05 but differ dramatically in clinical importance depending on their effect size. Conversely, an effect with *p* = 0.051 may still be clinically relevant if its magnitude is substantial [3, 7]. Confidence intervals complement this by conveying both direction and precision, allowing more nuanced interpretation than a binary threshold.

The movement away from dichotomous decision‐making toward estimation‐based inference aligns directly with contemporary Open Science principles. Transparency, reproducibility, and cumulative evidence synthesis, which are central pillars of Open Science, depend on continuous measures of effect and uncertainty rather than binary thresholds [8]. Estimation encourages more informative replications and clarifies the inherent variability of study outcomes, supporting a more accurate and self‐correcting scientific process [4]. In parallel, methodological initiatives that advocate moving ‘beyond *p* < 0.05’ reinforce the need to abandon arbitrary cutoffs and instead communicate compatibility, magnitude, and contextual uncertainty [9]. Integrating these perspectives situates statistical inference within a broader framework of scientific integrity and open research practices while simultaneously strengthening cumulative science by promoting more informative replications and enabling meta‐analyses grounded in effect estimates rather than binary significance testing.

In sports and clinical research, these concerns are especially acute. Many ‘significant’ findings are based on underpowered studies, prone to false positives and questionable research practices such as *p*‐hacking or HARKing [10, 11]. The disproportionate reliance on *p* values has therefore undermined credibility, inflated publication bias, and slowed scientific progress.

In this context, it is imperative to reconsider the central role of the *p* value. The present review critically examines methodological literature that challenges the *p* value paradigm and synthesizes proposed alternatives, including effect sizes, confidence intervals, Bayesian approaches, and emerging tools for representing uncertainty. Our aim is to provide researchers in health and sports sciences with a clearer framework for interpreting evidence beyond dichotomous thresholds, highlighting strategies that enhance transparency, reproducibility, and clinical relevance.

## 2 Methods

### 2.1 Study Design

This study was conducted as a critical review, a form of narrative synthesis that goes beyond a descriptive summary of the literature to provide an interpretative and critical analysis of available evidence, often generating new perspectives or theoretical hypotheses [12]. This type of review emphasizes conceptual integration and depth of discussion and is recognized as a legitimate method of biomedical knowledge synthesis [13]. The quality of narrative reviews can be assessed with structured tools such as the Scale for the Assessment of Narrative Review Articles (SANRA) [14], which reinforces their methodological relevance and contribution to scientific communication and clinical practice.

### 2.2 Search Strategy

We deliberately restricted the search to PubMed/MEDLINE, given its comprehensive biomedical coverage and controlled vocabulary (MeSH), which enhances precision and standardization of bibliographic retrieval [15]. Two reviewers independently conducted the search, with results cross‐checked to reduce selection bias. The strategy combined terms related to hypothesis testing and statistical significance (“*p*‐Value”[MeSH], “Hypothesis Testing”[MeSH], “Statistical Significance”[MeSH], “*p* value”) with descriptors for alternative metrics (“Effect Size”[MeSH], “Confidence Intervals”[MeSH], “Bayesian Analysis”[MeSH]). Filters were applied to restrict results to reviews published between 2015 and 2025.

### 2.3 Inclusion and Exclusion Criteria

We included articles explicitly addressing limitations of the *p* value and/or presenting alternative metrics such as effect sizes, confidence intervals, Bayesian approaches, magnitude‐based inference, or the fragility index. Eligible publications comprised reviews, methodological essays, tutorials, guidelines, editorials (e.g., ASA Statement 2016/2019), and scholarly commentaries with substantive methodological content. Empirical studies were included only when their primary aim was methodological (e.g., introducing or testing the fragility index, or comparing *p* values with alternative approaches). Citation chasing (snowballing) was used to capture highly cited classical works prior to 2015, ensuring adequate historical context.

Exclusion criteria were applied to articles reporting *p* values without methodological discussion, purely technical mathematical work without clinical or sports relevance, empirical studies not focused on methodological issues, animal or laboratory research without applied implications, conference abstracts, short letters, and duplicates.

### 2.4 Screening and Data Extraction

After deduplication in Rayyan [16], titles and abstracts were screened independently by two reviewers. Disagreements were resolved by consensus. Citation chasing was performed for key included papers. From eligible articles, we extracted bibliographic data (author, year, journal), methodological focus (*p* value, effect size, confidence intervals, Bayesian approach, magnitude‐based inference, fragility), key definitions and arguments, practical recommendations, applied context (sports science or biomedicine), and quality indicators. Extracted data were summarized in a table.

### 2.5 Quality Appraisal

Methodological quality was assessed with SANRA [14], focusing on topic relevance, clarity of objectives, search description, reference appropriateness, scientific grounding, and data presentation. For editorials and position papers, a simplified checklist was applied, assessing clarity of thesis, use of literature, and coherence of recommendations. All appraisals were independently conducted by two reviewers, and any discrepancies were resolved through consensus.

### 2.6 Critical Appraisal Tool

We also applied a bespoke 5‐point Likert‐type instrument (adapted [17]), developed specifically for this study, covering six methodological dimensions: (i) explicit critique of *p* value use; (ii) proposal of alternative metrics (e.g., effect sizes, confidence intervals, Bayesian approaches, *S*‐values); (iii) integration with clinical or practical relevance; (iv) discussion of fragility and reproducibility (e.g., preregistration, open data, statistical power); (v) suggestion of a paradigm shift; and (vi) use of empirical evidence or concrete examples. Each item was scored from 0 (absent) to 4 (detailed with empirical examples), for a total of 0–24 points. Scores were further categorized as weak (0–8), moderate (9–16), or robust (17–24).

Assessments were independently conducted by two trained reviewers using a written rubric with operational anchors. Discrepancies were resolved through consensus, and justifications for final scores were recorded for auditability. Results were summarized descriptively using medians and interquartile ranges (P25–P75). Bar charts illustrated the distribution of scores by domain. No hypothesis testing was performed. Analyses were carried out in R (v.4.5.1) with RStudio (v.2024.4.2.764).

## 3 Results

**Figure 1:**
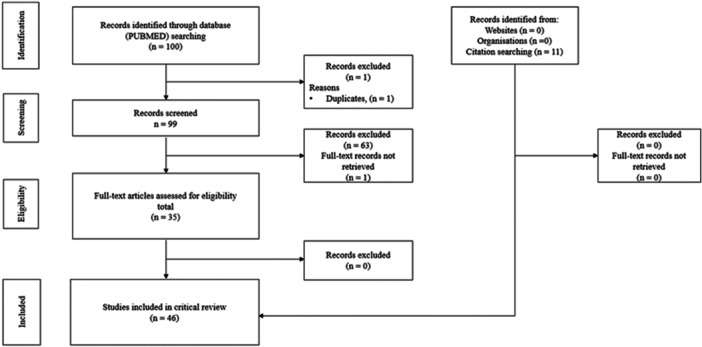
Flow of identification, screening, eligibility, and inclusion of studies adapted from the PRISMA diagram for critical review.

The PubMed/MEDLINE search retrieved 100 records. After deduplication, one duplicate was identified, leaving 99 unique articles. Of these, 63 were excluded during screening for not meeting the predefined methodological scope. Thirty‐seven articles were retrieved for full‐text reading, of which one was inaccessible. Thus, 36 articles were included from the systematic search, supplemented by 11 additional studies identified through citation chasing, resulting in a total of 46 included papers. A PRISMA flow diagram [18] was adapted to illustrate the selection process for this critical review (Figure 1).

The extracted results from each study are presented in Tables 1 and 2.

**Table 1:** Characteristics of articles included through citation chasing (snowballing).

Year/Authors	Focus/Limitations	Recommendations	Context
[19]	Problems with frequentist methods; *p* value overestimates evidence; does not reflect probability of the null hypothesis.	Use Bayes factor as a measure of evidence; integrate prior knowledge.	Medical statistics; clinical research.
[20]	Poorly supported medical conclusions; *p* value creates an illusion of objectivity.	Use Bayes factor and CIs for evidence integration.	Clinical trials; medical research.
[21]	Criticism of *p* = 0.05; misinterpretation; positive findings published as if they were strong.	Interpret within context; use CIs (preferably 90%); stop treating *p* < 0.05 as special.	Medical and epidemiological research.
[22]	Criticism of the ‘null ritual’ and mixing of theories; *p* value does not convey probability of a hypothesis.	Prioritize descriptive statistics; publish exact significance; avoid dichotomies.	Psychology; social sciences; experimental research.
[23]	Many false findings; bias, small studies, and multiple testing increase false results.	Improve study power; use meta‐analyses; preregistration; reduce bias.	Biomedical research, epidemiology, and genomics
[24]	NHST does not provide magnitude or precision; encourages dichotomies.	Report effect sizes + CIs; prioritize non‐standardized ES; useful for meta‐analyses.	Biomedical research, epidemiology, and genomics.
[25]	Misinterpretation of the *p* value; does not measure the probability that a conclusion is correct; overestimates evidence.	Consider effect size and full CIs; adopt Bayesian measures.	Medical research; clinical trials
[26]	Low power → false findings; winner's curse; *p‐*hacking increases false positives.	Calculate a priori power; preregister; share data; increase collaborations.	Neuroscience; biomedical research.
[27]	Researchers misinterpret effect sizes; incorrect use of partial eta‐squared.	Preregistration; two‐step analysis; distinguish exploratory from confirmatory analyses.	Psychology; social sciences.
[28]	Researchers misinterpret effect sizes; incorrect use of partial eta‐squared.	Always report ES (with subscripts); use generalized eta‐squared; conduct power analyses.	Experimental psychology; ANOVA.
[29]	*p* values are not objective; *p*‐hacking increases false positives; many false findings.	Report ES + CIs; use Bayesian approaches; full transparency; preregistered replications.	Biomedical sciences; psychology; epidemiology.

Abbreviations: ANOVA, Analysis of Variance; CIs, Confidence Intervals; ES, Effect Size; NHST, Null Hypothesis Significance Testing.

**Table 2:** Characteristics of articles included through the systematic PubMed/MEDLINE search (2015–2025).

Year/Authors	Focus/Limitations	Recommendations	Context
[30]	A threshold of *p* ≤ 0.05 is often misinterpreted as rigid; ritualistic use persists; NHST is banned in some journals.	Interpret strength and stability with ES + CI; avoid the *p* ritual.	Clinical investigations; genomics.
[31]	ASA warned about misuse; *p* does not measure effect/importance; frequentist methods unsatisfactory.	Transparent reporting; consider Bland–Altman method for comparisons.	Statistics in medicine; orthodontics.
[32]	*p* and CI based on the same mathematics; *p* does not measure strength/power; impractical in high‐throughput biology.	Comprehensive synthesis of results; avoid isolated tests; emphasize uncertainties.	Statistical methodology; scientific research.
[33]	*p* and CI based on the same mathematics; p does not measure strength/power; impractical in high‐throughput biology.	Focus on estimated ES + CI; *p* only auxiliary; use replication/resampling.	Statistics; high‐throughput biology.
[34]	Volatility of the *p* value; results vary widely even in ideal experiments; unreliable as a single criterion.	Focus on ES and CI as estimates of magnitude/precision; interpret p as continuous; use replication/resampling.	Applied statistics in biomedicine and biological sciences.
[35]	Large n → small effects appear significant.	Focus on ES + CIs; clinical relevance should guide practice.	Otolaryngology; clinical research.
[36]	*p* fails to show the full effect; excessive focus on significance.	Use CIs for magnitude; focus on clinical relevance; accept uncertainty.	Medicine; clinical trials.
[37]	Arbitrary 0.05 cutoff; promotes *p*‐hacking; significance depends on *n*.	Interpret 95% CI according to clinical relevance; classify findings by clinical impact.	Medicine; clinical trials.
[38]	Dependence on NHST; wide CIs due to small samples.	Report ES + CI; conduct power analyses; report variability measures.	Experimental psychology; behavioral sciences.
[39]	Misuse of *p*; any effect can be ‘significant’ with large n.	Translate ASA warnings; use CIs for magnitude; apply NNT.	Nephrology; clinical trials.
[40]	*p* < 0.05 overreported; CI underused; power analyses rare.	Plan tests; report ES + CI instead of isolated *p*.	Health education; biomedical research.
[41]	*p* < 0.05 in observational studies ≈ 50% false positive.	Highlight ES + CI; evaluate clinical relevance in context.	Observational studies; epidemiology.
[42]	Errors in articles; *p* suggests undue significance; reproducibility threatened.	Identify and mitigate errors; strengthen peer review.	Epidemiology; MEDLINE abstracts.
[43]	Weak adherence to CI reporting in multivariable regressions; p not in STROBE.	Follow STROBE; reinforce through editorial policies.	Observational studies MEDLINE; epidemiology.
[44]	Abuse and misinterpretations; p does not indicate magnitude; incentivizes *p*‐hacking.	Use stricter threshold (*p* < 0.005); report ES + CI; larger samples; adopt term “suggestive” for *p* < 0.05.	Biomedical sciences; ophthalmology.
[45]	Isolated p does not show effect; CIs not always adequate in descriptive studies.	Report ES + CIs as standard; editors should require.	Plastic surgery; medical statistics.
[46]	*p* < 0.05 seen as “grail”; incentive for manipulation.	Require ES + CI in publications; consider effect direction.	Spine surgery; medical statistics.
[47]	*p* is not a sole guide; paradigm shift difficult.	Publish negative results; reviewers should require careful interpretation.	Plastic surgery; scientific publishing.
[48]	Incorrect use of p with fixed threshold; credibility compromised; technique never neutral.	Avoid ‘statistically significant’; report estimates with 90% CI; consider clinically relevant difference a priori.	Epidemiology; scientific research.
[49]	NHST and dichotomous p → reproducibility crisis; CI misinterpreted.	Prioritize estimation; use *p* value functions; counternull.	Medical research; statistical inference.
[50]	CI alone not useful for clinical decisions.	Use meta‐analyses to narrow CI; focus on clinical relevance.	Physiotherapy; meta‐analysis.
[51]	Misinterpretations persist; CI containing null = ‘no benefit.’	Reinterpret CIs as compatibility; use S‐values.	Medicine; statistical methodology.
[52]	Cognitive and technical problems; pressure for definitive conclusions; harmful dichotomy.	Replace terms with ‘compatibility’; complement *p* with S‐values; present results for multiple hypotheses.	Medical research; statistical science; registry‐based cohort.
[53]	Isolated *p* does not show full picture; confounded by n and variability.	Report CIs + exact *p*; include absolute ES and Cohen's *d*.	Medical research; graduate theses.
[54]	Inadequate reliance on the *p* value as the central metric, with the traditional 0.05 cutoff lacking a scientific basis. Common misinterpretations include assuming that smaller *p* values indicate larger effects. In addition, surgical studies involving rare events often face challenges in achieving the conventional threshold of *p* < 0.05.	Always report effect sizes and confidence intervals; avoid terms like ‘trend toward significance’; consider raising significance threshold to 0.1 in specific contexts (rare events, low risk, serious clinical implications); encourage publication of negative results; reviewers and editors should interpret data based on study design, not just *p*	Plastic surgery; biomedical literature; methodological discussion applied to clinical trials and meta‐analyses.
[55]	Excessive dependence on *p* in biomedical research; problematic dichotomization.	Use *p* value function and drapery plot in meta‐analyses.	Meta‐analyses; ecology; biomedicine.
[56]	*p* values and statistical significance often misinterpreted; ‘not significant’ ≠ no effect; low power creates uncertainty disguised as certainty.	Interpret *p* as a continuous measure; consider effect size and confidence intervals; explicitly discuss uncertainty; avoid ‘significant/non‐significant’ dichotomy.	Editorial in orthopedic surgery/arthroscopy; applied statistics in clinical research.
[57]	Misuse of *p*; arbitrary cutoff; *p* does not measure magnitude.	Promote ES + CI as primary; *p* only complementary.	Cardiology; scientific research.
[58]	Misleading statistical reporting in health; excessive focus on ‘significance/confidence’; dichotomization neglects uncertainties.	Use ‘compatibility’ instead of significance/confidence; no cutoff for *p*; compatibility intervals; challenge editorial conventions.	Medical journals; health statistics; clinical trials.
[59]	Multiple hypotheses inflate type I error; CIs under‐corrected.	Apply corrections (Bonferroni, Holm, Romano–Wolf); use permutation.	Cluster‐randomized clinical trials.
[4]	NHST default despite criticisms; *p* unreliable; replicability crisis.	‘Estimation first’: ES + CI + meta‐analysis; Open Science; quantitative hypotheses.	Psychology; neuroscience; behavioral sciences.
[60]	*p* = 0.05 does not reflect clinical relevance; *p* misinterpreted; CI misunderstood.	Report ES + CI; use MCID; consider effect magnitude and direction.	Anesthesiology; pain; clinical trials.
[61]	Focus on *p* reduces sample size for significance; use of ‘trend statements.’	Define clinically relevant outcomes; optimize sample sizes.	Oncology; clinical trials.
[62]	Need to compare RCTs and RWE; risk of heterogeneous estimates.	Standardize RWE protocols; meta‐analysis + skeptical *p*.	RCTs; RWE emulation.
[63]	CIs provide magnitude, precision, and clinical relevance of effect but are underused and often misinterpreted. *p* value does not indicate effect size or importance.	Always report effect + CI (95%/99%); make editorial requirement; complement or replace *p* value with estimation methods (CI, Bayes, *p* value functions). Interpret considering null value and clinical relevance.	Methodological article in medical statistics (Reviews on Recent Clinical Trials, 2024), focused on evidence‐based clinical research.

Abbreviations: ASA, American Statistical Association; CI, Confidence Interval; ES, Effect Size; MCID, Minimum Clinically Important Difference; NHST, Null Hypothesis Significance Testing; NNT, Number Needed to Treat; RCT, Randomized Controlled Trial; RWE, Real‐World Evidence; STROBE, Strengthening the Reporting of Observational Studies in Epidemiology.

Of the 46 included studies, 38 (82.6%) presented direct criticism of the isolated or dichotomous use of the *p* value (“significant/non‐significant”), highlighting problems related to interpretation, reproducibility, and clinical relevance [4, 19, 20, 22, 23, 25, 26, 27, 29, 30, 32, 34, 35, 36, 37, 38, 39, 40, 41, 42, 43, 44, 45, 46, 47, 48, 49, 51, 52, 53, 54, 55, 56, 58, 59, 60, 62, 63].

**Figure 2:**
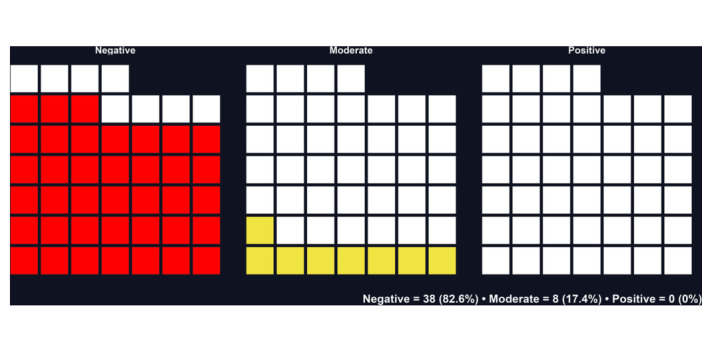
Overall stance of included studies toward the use of *p* values. Waffle plot illustrating the distribution of the 46 included studies according to their position on the use of *p* values: negative (*n* = 38; 82.6%), moderate (*n* = 8; 17.4%), and positive (*n* = 0; 0%). Each square represents one study.

Eight studies (17.4%) expressed a more moderate critique, acknowledging important limitations but still admitting some use of the *p* value in specific contexts or when combined with other metrics [21, 24, 28, 31, 33, 50, 57, 61]. None of the included studies endorsed the use of the *p* value alone as a criterion for scientific decision‐making. These data are illustrated in the waffle plot (Figure 2), in which each square represents an included article (*n* = 46). The plot shows that most studies adopted a negative stance toward the isolated use of the *p* value, while a smaller proportion adopted a moderate position, and none supported its use positively (Figure 2).

### 3.1 Methodological Quality Assessment

The narrative reviews included in this study were evaluated using the Scale for the Assessment of Narrative Review Articles (SANRA) [14], which comprises six items: (1) justification and importance of the topic; (2) clarity of objectives; (3) description of the literature search process; (4) adequacy, currency, and presentation of references; [14] scientific reasoning and critical analysis; and (6) presentation of relevant and appropriate outcome data. Each item is scored from 0 to 2, yielding a maximum total score of 12 points.

A total of 25 articles were assessed with SANRA [14]. The lowest score observed was 8/12 and the highest 11/12. Specifically, 6 articles (23.1%) scored 8, 5 (19.2%) scored 9, 8 (30.8%) scored 10, and 7 (26.9%) achieved 11/12. No study reached the maximum score of 12/12. Among the six items, item 3 (description of the literature search process) performed worst: none of the studies achieved the maximum (2/2), 50% scored 0/2, and the remaining 50% scored 1/2 (Table 3).

**Table 3:** Methodological quality assessment of included narrative reviews using the SANRA tool.

Author/Year	Importance	Objectives	Search	Referencing	Reasoning	Data	Score
[19]	2	2	1	2	2	1	10
[20]	2	2	1	2	2	1	10
[21]	2	2	0	2	2	1	9
[22]	2	2	0	2	2	2	10
[23]	2	2	1	2	2	2	11
[24]	2	2	1	2	2	2	11
[25]	2	2	1	2	2	1	10
[26]	2	2	1	2	2	2	11
[27]	2	2	1	2	2	1	10
[30]	2	2	0	1	2	1	8
[32]	2	2	1	2	2	1	10
[36]	2	2	0	1	2	2	9
[37]	2	2	0	1	2	2	9
[39]	2	2	0	1	2	2	9
[40]	2	2	0	1	2	1	8
[46]	2	2	0	1	2	1	8
[49]	2	2	1	2	2	2	11
[50]	2	2	0	1	2	2	9
[51]	2	1	0	2	2	1	8
[52]	2	2	1	2	2	2	11
[53]	2	2	0	1	2	1	8
[55]	2	2	1	2	2	2	11
[58]	2	2	1	2	2	1	10
[4]	2	2	1	2	2	2	11
[60]	2	2	0	2	2	2	10
[61]	2	2	0	1	2	1	8

### 3.2 Critical Appraisal Using the Bespoke Instrument

The critical appraisal of the 46 included articles was conducted with the bespoke Likert‐type instrument (0–4 points) [17] developed for this study, covering six methodological dimensions: (i) explicit critique of *p* value use; (ii) proposal of alternative metrics; (iii) practical or clinical relevance; (iv) reproducibility; (v) suggestion of a paradigm shift; and (vi) use of empirical evidence.

**Figure 3:**
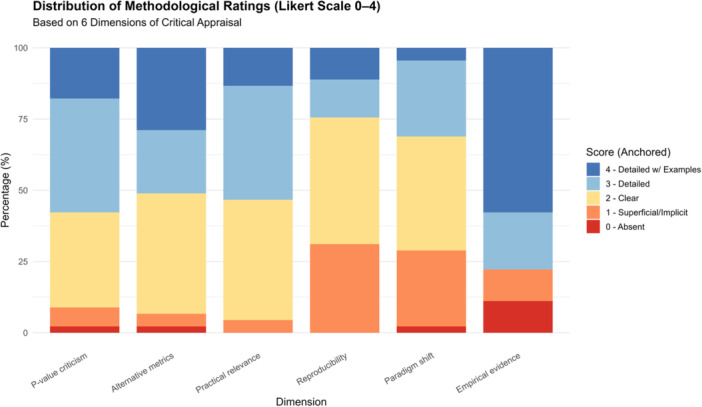
Distribution of methodological ratings across six dimensions of critical appraisal. Stacked bar chart showing the percentage of scores (0–4 Likert scale) across the six methodological dimensions assessed: *p* value criticism, alternative metrics, practical relevance, reproducibility, paradigm shift, and empirical evidence. Higher scores (3 = *detailed*; 4 = *detailed with examples*) were more frequent in the domains of *p* value criticism and alternative metrics, while reproducibility and paradigm shift showed higher proportions of lower scores (0–1).

The percentage distribution of scores across dimensions is shown in Figure 3. The *p* value criticism and Alternative metrics dimensions concentrated the highest proportions of scores at levels 3 (detailed) and 4 (detailed with examples). In contrast, Reproducibility and Paradigm shift presented higher frequencies of scores at lower levels (0 = *absent* and 1 = *superficial/implicit*). The Empirical evidence dimension stood out as the most consistent, with a predominance of level 4 scores, indicating frequent use of practical examples and empirical applications (Figure 3).

**Figure 4:**
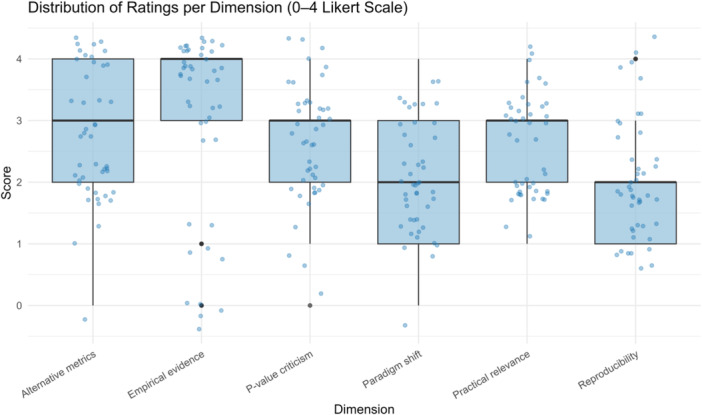
Distribution of ratings per dimension (0–4 Likert scale). Boxplots display the interquartile range, with the thick horizontal line representing the median. Whiskers extend to adjacent values within 1.5 × IQR, and points beyond the whiskers indicate potential outliers. Alternative metrics and empirical evidence received the highest median ratings (≥ 3), whereas reproducibility received the lowest median (2) and showed the widest dispersion.

In addition, Figure 4 displays the distribution of individual scores by dimension in boxplot format. Alternative metrics and Empirical evidence showed the highest medians (≥3 points), while Reproducibility presented the lowest median (2 points) and the greatest dispersion, suggesting variability among studies. Overall, the pattern confirms that most articles provide detailed critiques of *p* value use and propose robust methodological alternatives, whereas reproducibility issues remain insufficiently addressed.

## 4 Discussion

This critical review demonstrates a consistent body of methodological literature emphasizing the limitations of relying on the *p* value as the central criterion for statistical inference. The main criticism and proposed alternatives identified across the included studies are summarized in Box 1. Most studies adopt a negative stance toward the isolated use of statistical significance, stressing that such practice oversimplifies complex data, reinforces artificial dichotomies, and undermines reproducibility. A smaller subset of papers acknowledged that *p* values may retain some complementary value when interpreted alongside effect sizes, confidence intervals, or Bayesian methods. Importantly, no study supported the use of the *p* value alone as a sufficient basis for scientific or clinical decision‐making. These findings reinforce international calls to move beyond the “significant/non‐significant” dichotomy and underscore the importance of adopting more informative approaches to evidence appraisal in health and sports sciences.

A recurring recommendation across the reviewed literature is the systematic use of effect sizes (ES) as the central metric for statistical interpretation. Unlike the *p* value, ES provides information on the actual magnitude of an association or difference, allowing researchers and clinicians to distinguish between trivial and clinically meaningful results, even when statistical significance is achieved. Commonly reported measures include mean differences, relative risk, odds ratio, absolute risk reduction, Cohen's *d*, and generalized eta‐squared, each adapted to specific research contexts. Authors such as Goodman [25], Nakagawa & Cuthill [24], Lakens [28], and Morris and Fritz [38] have emphasized that effect sizes should be consistently reported and accompanied by measures of variability to serve as a foundation for scientific inference and clinical decision‐making. This recommendation is echoed in major international guidelines: the CONSORT 2010 Statement requires reporting of effect estimates with confidence intervals in clinical trials [64], the APA Publication Manual mandates effect size reporting in psychological and experimental research, and editorials in The American Statistician [6] and the New England Journal of Medicine (2019) [65] have urged the field to move beyond the “statistical significance” paradigm. Together, these calls reinforce that effect sizes are not optional complements but essential metrics for transparent and clinically relevant interpretation.

Confidence intervals (CIs) have emerged as another widely advocated alternative, as they provide not only a point estimate but also the plausible range of values for the true effect, simultaneously conveying precision, direction, and plausibility. Critics such as Sterne and Smith [21], Ranganathan et al. [2], and Martínez‐Ezquerro et al. [37] have argued that CIs should be interpreted contextually, particularly in relation to clinical relevance (e.g., the minimal clinically important difference, MCID), rather than simply by whether they include the null value. Some authors have proposed reporting 90% CIs to enhance practical interpretability, while others emphasize abandoning the dichotomous reading of ‘contains/does not contain the null.’ Importantly, this perspective aligns with major reporting guidelines such as the CONSORT Statement for clinical trials [64] and the STROBE Statement for observational studies [66], which explicitly require effect estimates to be reported with confidence intervals. Furthermore, recent editorials in The American Statistician [6] and the New England Journal of Medicine (2019) [65] have reinforced that CIs should be interpreted as compatibility intervals that reflect the range of effect sizes most consistent with the data, rather than as dichotomous tests. Together, these positions consolidate CIs as central tools for replacing the binary logic of statistical significance with a more nuanced, clinically meaningful interpretation of evidence. Some authors have also emphasized hypothesis evaluation using an interval null, which avoids binary decisions by assessing compatibility with a range of practically null effects rather than a point null.

Bayesian approaches have been increasingly recognized as coherent alternatives to frequentist inference, as they allow explicit incorporation of prior knowledge into the evaluation of evidence. The use of Bayes factors, for instance, has been advocated by Goodman [20], Goodman [25], and Greenland et al. [32] as a more interpretable way of expressing the strength of evidence for or against a hypothesis compared to *p* values. Beyond hypothesis testing, Bayesian hierarchical and probabilistic models enable direct quantification of the probability of parameters or hypotheses, thus overcoming key limitations of frequentist inference. Recent comprehensive reviews in sports science highlight the growing adoption of Bayesian methods to model complex problems, integrate multiple sources of information, and deal effectively with small datasets—contexts in which traditional approaches often fail [67]. Applied examples illustrate this potential: Deshpande and Jensen [68] employed Bayesian regression to estimate NBA players' contributions to their teams' win probabilities, showing how posterior distributions can provide richer insights than point estimates. Collectively, these studies reinforce that Bayesian approaches are not only statistically rigorous but also particularly valuable in applied biomedical and sports contexts, where uncertainty, prior knowledge, and small sample sizes are common.

A more recent line of proposals recommends reinterpreting *p* values through alternative forms of presentation. *p* value functions graphically display the levels of compatibility of the data with different hypotheses, while *S*‐values express the ‘surprise’ associated with a result in a more intuitive metric. More recently, Cole et al. [51], Rafi and Greenland [52], and Rücker and Schwarzer [55] have advocated *p* value functions and S‐values as effective means of moving beyond the significant/non‐significant dichotomy toward graded, uncertainty‐aware interpretations of evidence. The use of these visual and mathematical representations allows uncertainty to be communicated more transparently, particularly in the context of meta‐analyses, where heterogeneity between studies plays a central role. In this regard, the work of IntHout et al. [69] demonstrated that small studies tend to be more heterogeneous than larger ones, underscoring the importance of approaches that convey compatibility and variability rather than relying exclusively on arbitrary thresholds of statistical significance.

In addition to these core proposals, some studies have highlighted alternative indicators aimed at enhancing clinical interpretation and robustness of findings. The Number Needed to Treat (NNT) has long been advocated as a clinically meaningful measure to quantify the impact of interventions, with seminal contributions by Laupacis et al. [70] and methodological refinements by Altman [71], who emphasized the importance of reporting confidence intervals alongside NNT. The Fragility Index, originally introduced to assess how many events would need to change status to overturn the statistical significance of a trial, has further exposed the vulnerability of many randomized controlled trials [72, 73]. Finally, the Drapery Plot has been proposed as a valuable complement to the traditional forest plot in meta‐analyses, as it displays confidence intervals across multiple significance levels, offering a more nuanced visualization of uncertainty [55]. While less universal than effect sizes or confidence intervals, these indicators contribute to a richer and more multifaceted appraisal of evidence, helping to move beyond the reductionism of the isolated *p* value.

In summary, the findings of this review reinforce that the central role historically attributed to the *p* value does not meet contemporary demands for rigor, transparency, and clinical relevance. The literature converges on the view that scientific interpretation cannot be reduced to an arbitrary threshold, but should incorporate more informative metrics such as effect sizes, confidence intervals, Bayesian approaches, *p* value functions, and robustness indicators. Shifting the focus from the ‘significant/non‐significant’ dichotomy toward the evaluation of magnitude, precision, and plausibility offers a stronger path to address the reproducibility crisis and to promote higher‐quality evidence‐based decision‐making. This perspective aligns with recent international statements from the American Statistical Association [6, 9] and leading journals such as the New England Journal of Medicine (2019) [65], which have called for retiring the misuse of statistical significance. Ultimately, overcoming overreliance on the *p* value is not only a methodological concern, but a fundamental step toward aligning biomedical and sports research with its overarching purpose: producing knowledge that is reliable, applicable, and clinically meaningful. These methodological shifts are consistent with broader Open Science practices, which emphasize transparency, reproducibility, preregistration, open materials, and cumulative evidence synthesis. Embedding statistical reform within Open Science principles further strengthens the credibility and societal impact of scientific research [74].

This review has several limitations. First, the search strategy relied exclusively on the PubMed/MEDLINE database, which, although justified by its comprehensive biomedical coverage and controlled vocabulary (MeSH), may have excluded relevant publications from other databases. Second, the inclusion of classic works through citation tracking was not based on a fully explicit or systematic procedure, which may have introduced selection bias. Third, the critical appraisal instrument developed specifically for this study, despite being anchored and applied by independent reviewers, has not undergone prior validation, which limits the generalizability of its scores. Finally, as a narrative critical review, this study is inherently subject to limitations such as the absence of a preregistered protocol and potential subjectivity in evidence synthesis, even though efforts were made to increase transparency through predefined rubrics, independent assessment, consensus resolution, and detailed reporting.

Box 1 **Practical recommendations for moving beyond the *p* value dichotomy.**

- Pose quantitative research questions (“to what extent…?”).
- Report effect sizes with compatibility intervals as primary results.
- Interpret uncertainty explicitly; avoid dichotomous terms.
- Use estimation‐focused graphics (e.g., Gardner–Altman, drapery plots, p value functions).
- Preregister hypotheses and analysis plans.
- Adopt cumulative reasoning (meta‐analytic thinking).
- Perform robustness and sensitivity analyses.
- When applicable, evaluate hypotheses using an interval null.
- Share data, materials, and code (Open Science practices).
- For clinicians: emphasize magnitude and precision rather than thresholds.

## 5 Conclusion

In summary, this review highlights the urgent need to move beyond the binary interpretation of *p* values in scientific inference. The evidence consistently shows that isolated statistical significance offers limited insight into effect magnitude, precision, and clinical or practical meaning. Replacing it with estimation‐based reasoning—using effect sizes, compatibility intervals, and Bayesian approaches—fosters more transparent, reproducible, and decision‐relevant research. Abandoning the “significant/non‐significant” dichotomy and embracing continuous, context‐aware metrics is therefore essential to strengthen methodological rigor and align statistical practice with the real objectives of biomedical and sports sciences.

## Funding

The authors received no specific funding for this work.

## Ethics Statement

Ethical approval was not required for this study, as it is a critical review of published literature.

## Conflicts of Interest

The authors declare no conflicts of interest.

## Data Availability

The data that support the findings of this study are available from the corresponding author upon reasonable request.
